# Optimization of Microwave-Based Heating of Cellulosic Biomass Using Taguchi Method

**DOI:** 10.3390/ma6083404

**Published:** 2013-08-09

**Authors:** Kuo-Hsiung Tseng, Yong-Fong Shiao, Ruey-Fong Chang, Yu-Ting Yeh

**Affiliations:** 1 Department of Electrical Engineering, National Taipei University of Technology, 1, Sec. 3, Chung Hsiao E. Rd., Taipei 10608, Taiwan; E-Mail: ntutee219@gmail.com; 2 GTI-GreenTech International Co., Ltd., Taipei 10608, Taiwan; E-Mail: greentech313@yahoo.com.tw

**Keywords:** microwave-based heating, biomass material, pretreatment, taguchi method, *Pennisetum purpureum*, system Identification

## Abstract

This study discusses the application of microwave-based heating for the pretreatment of biomass material, with *Pennisetum purpureum* selected for pretreatment. The Taguchi method was used to plan optimization experiments for the pretreatment parameter levels, and to measure the dynamic responses. With a low number of experiments, this study analyzed and determined a parameter combination in which *Pennisetum purpureum* can be rapidly heated to 190 °C. The experimental results suggested that the optimal parameter combination is: vessel capacity of 150 mL (level 2), heating power of 0.5 kW (level 1), and mass of *Pennisetum purpureum* of 5 g (level 1). The mass of *Pennisetum purpureum* is a key factor affecting system performance. An eight-order ARX model (Auto-Regressive eXogeneous) was representative of the actual system performance, and the fit was 99.13%. The results proved that microwave-based heating, with the assistance of the Taguchi method for pretreatment of the biomass material, can reduce the parameter combination variations.

## 1. Introduction

Lignocellulosic biomass is one of the most abundant and clean resources in the world that can be converted to bio-ethanol [1,2]. The steps for converting cellulose from biomass waste into alcohol include pretreatment, hydrolysis, and alcoholic fermentation, where pretreatment is a key step. Lignocellulosic material pretreatment methods can be roughly divided into four categories: (1) physical pretreatment; (2) chemical pretreatment; (3) physical/chemical pretreatment; and (4) biological pretreatment. Physical pretreatment can be divided into mechanical grinding and cracking; physical/chemical pretreatment can be divided into the Steam Explosion and Hydrothermal Method, Ammonia Explosion and CO_2_ Explosion; chemical pretreatment can be divided into acid treatment, alkali treatment, the oxidative delignification treatment method with organic solvents; biological pretreatment uses degraded lignin for the pretreatment. Many studies have explored this for bio-ethanol. Steam explosion is a common pretreatment method for lignocellulose, a technology that was developed early in 1926 by W.H. Mason, who developed it to produce fiberboard and other products [3]. NREL (National Renewable Energy Laboratory), ENEA (Italian National Agency for New Technologies, Energy and Sustainable Economic Development), and CIEMAT (Centro de Investigaciones Energéticas, Medioambientales Tecnológicas) use the pretreatment technology of steam explosion to produce bio-ethanol [4]. Steam explosion is widely used as a pretreatment in current industrial production. The optimal conditions defined from experimental parameters can be used in the production process.

In traditional heating, heat energy is transferred from the outside of an object to the inside by means of thermal conductivity. As a result, more time is required, and objects with worse heat conductivity require longer time. On heating, a scorched outside, with an underdone inside, may easily occur. Microwave-based heating can simultaneously heat the outside and the inside; thus, heating effects can be achieved in a short time, and heating uniformity can be greatly improved [5]. For the purpose of economic efficiency, the pretreatment of lignocellulose, controlled by microwave-based heating, has been explored here.

Microwave-based heating was applied to the pretreatment of lignocellulose in early studies [3,6]. There are some current studies on the microwave-based treatment of biomass material [7,8]. In the literature [9], lignocellulose was placed in a closed vessel and heated in order to analyze the material. The results showed material could be decomposed and its structure remain undamaged when heated to 180 °C. Literature in recent years [10] has indicated that the pretreatment effect of biomass material is optimal when heated to 190 °C [11].

Many studies have compared traditional heating with microwave-based heating [12,13,14,15]. The results showed that microwave-based heating is quicker than traditional heating, and it has a better saccharification yield and efficiency. Looking at the above literature references, the use of microwave-based heating for the pretreatment of biomass material has great potential, is more rapid, and the relevant technologies are feasible.

In this study, the motivation for the study of *Pennisetum purpureum*, is because *Pennisetum purpureum* is a non-food crop, can be produced quickly in a high yield, and is not affected by pests, temperature and other factors. The goal of the study was to use the Taguchi method to find the optimal parameters, and system identification to establish a parametric model.

## 2. Methods

### 2.1. Microwave-Based Heating Device 

This study used a closed pressure vessel as a heat receiver, in which the microwave-based heating system consists of a microwave-based heating cavity and a control system. Four temperature sensors were installed to monitor temperature variations. The experiment employed the Taguchi method to determine the effect of the water volume of the closed vessel, the heating power and *Pennisetum purpureum* heating mass of the closed pressure vessel, in order to ascertain the optimal parameters of the factors, and to find out the effects of the parameters on the heating quality and variation in characteristics of the parameters.

After the optimal heating conditions were determined, the experiment was performed, and the input power and output (temperature) data were recorded. Next, the system identification method was used to construct the equation that conforms to the actual system.

**Figure 1:**
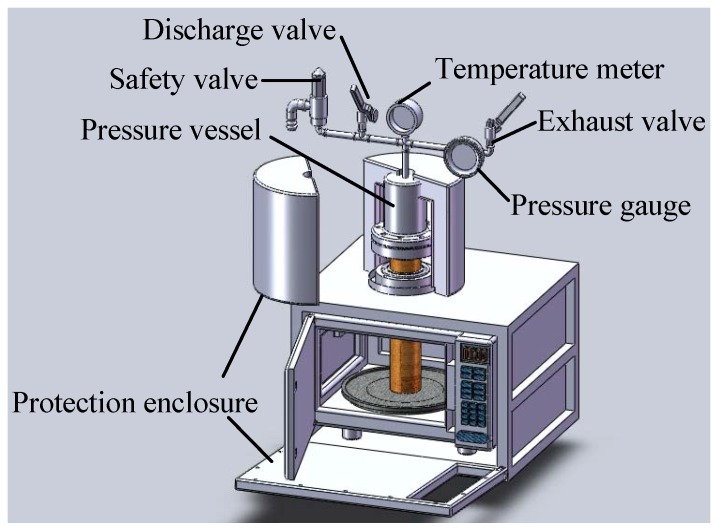
Microwave-based heating device and measuring equipment.

The microwave-based heating device used in this study is a SAMPO RE-115M type, which was modified as required by the experiment. The measuring equipment is shown in Figure 1.

### 2.2. Taguchi Method 

In pretreatment with steam explosion, the main operating conditions affecting the product composition include temperature and processing time [16], while improper treatment may affect the subsequent catalysis of the cellulase enzyme and the alcoholic fermentation [17]. This study heated lignocellulose (*Pennisetum purpureum*) to 190 °C [10], and used the optimal combination of parameters, as determined by the Taguchi method, to reduce the parameter combination variations.

**Figure 2:**
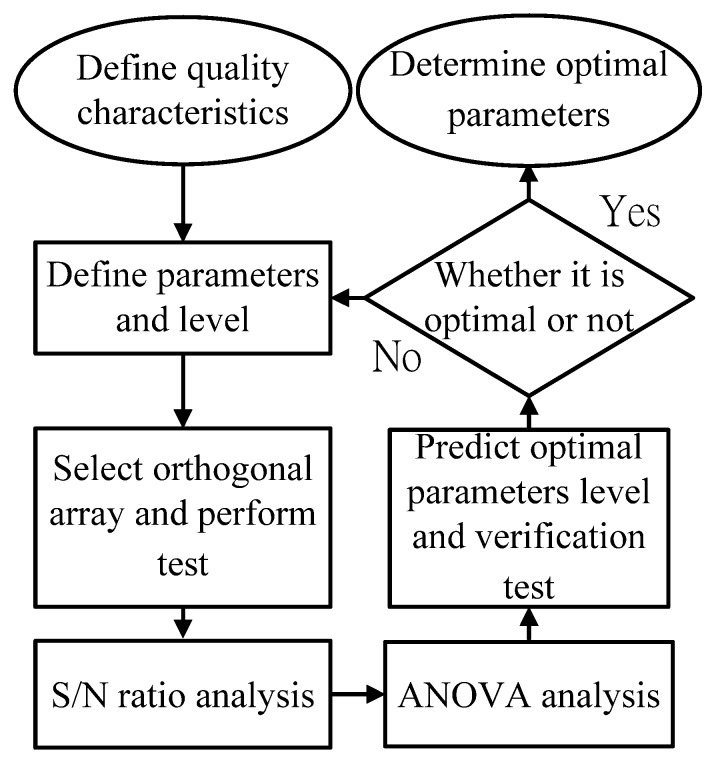
Taguchi method flow.

The Taguchi method can improve product quality by reducing the impact of factor variations, while using orthogonal arrays to reduce experiment frequency. Firstly, signal to noise ratio (*S/N*) is determined in terms of quality characteristics; secondly, *S/N* is used to evaluate the quality characteristics of different parameter combinations, and predict the optimal parameter combination; finally, verification experiments are conducted to show that the predicted combination can reduce the parameter variations and achieve the quality characteristics. The Taguchi method flow is as shown in Figure 2.

#### 2.2.1. Define Quality Characteristics 

In the experimental design of the Taguchi method, objective functions need to be identified to properly express the quality characteristics. This study shows how to make the vessel in a microwave-based heating device reach a specified temperature of 190 °C in the shortest time by changing the set parameters [10]. As required, it is better to heat the object to 190 °C with microwave-based heating within a short time. Thus, smaller-the-better is used as the method for calculating the experimental results of the quality characteristics. The *S/N* ratio for smaller-the-better is defined in Equation (1).

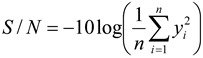
(1)
where, *y_i_* is the value of the *i* experiment in each group, and *n* is the frequency of experiment in each group.

**Figure 3:**
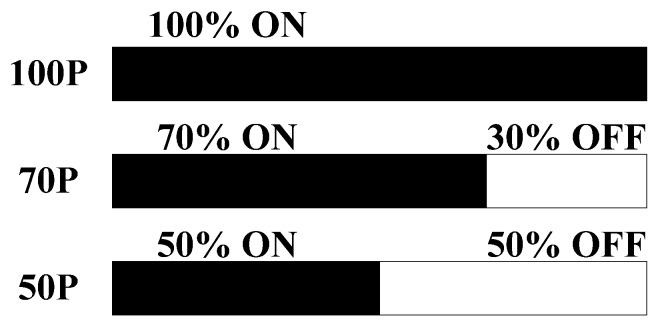
State diagram of microwave-based heating power.

The microwave-based heating device in this experiment is 1 kW, and the input power of the cavity is set to 100%, 70%, and 50%. The working state diagram is as shown in Figure 3.

#### 2.2.2. Define Parameters and Level 

**Figure 4:**
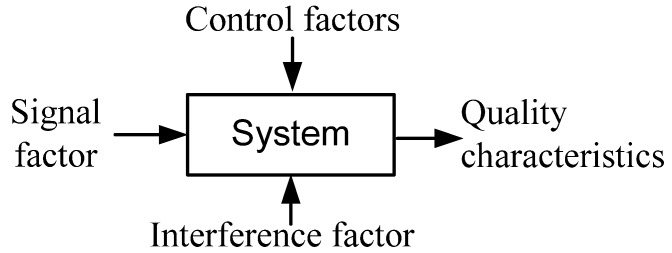
State diagram of microwave-based heating power.

For any product or process, a relational diagram of quality characteristics is plotted, as shown in Figure 4 [18,19], and such quality characteristics represent the response values to be discussed. The parameters affecting quality characteristics can be divided into signal factors, control factors, and interference factors. The signal factor and response value have input and output relations, and thus, the signal factor is the input of electric power; the control factor is an important factor of quality characteristics’ optimization. The control factors include the water volume of the vessel, heating power setting, and *Pennisetum purpureum* mass. The interference factor is an uncontrolled parameter, which cannot be determined for a special case, and is represented by heat loss. The parameter levels of the control factors in this study are as shown in Table 1.

**Table 1:** Level of control factor parameters.

Description	Level 1	Level 2	Level 3
Water volume of vessel (mL)	100	150	200
Power setting (kW)	0.5	0.7	1
*Pennisetum purpureum* mass (g)	5	10	15

### 2.3. System Identification 

**Figure 5:**
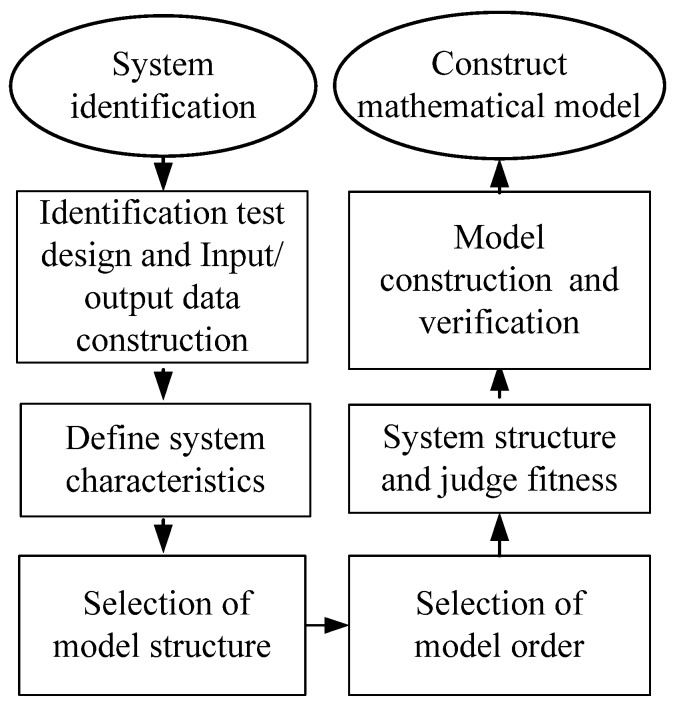
System identification flow chart.

The system identification inputs the signal of the actual system to deduce an approximate difference equation, or differential equation, to construct a system mathematical model. This study applied microwave-based heating to the pretreatment of biomass material, with water and *Pennisetum purpureum* in the vessel. If the model is constructed using a physical method, in addition to the electric power converted into the microwave gain, microwave distribution in the heating cavity, as well as the speed of water molecules absorbing microwave energy, should be considered. It is too complex to use a general differential equation for model construction; thus, the system has to be considered as a black box. Input and output signals were used to construct the approximate difference equation or differential equation through system identification [20,21]. The system identification flow is as shown in Figure 5.

## 3. Results and Discussion

### 3.1. Parameter Optimization Experiment Using the Taguchi Method

#### 3.1.1. Orthogonal Arrays 

With the orthogonal arrays in the Taguchi method, it is intended to use a low number of experiments to obtain useful statistical information. The representation method for orthogonal arrays is *La* (*n^w^*), where *L* is the Latin squares of the orthogonal array, *a* is the number of experiments, *n* is the number of factor levels, and *w* is the number of control factors. In selecting orthogonal arrays, the total degrees of freedom of the control factors must first be calculated. One factor has three levels, and the degree of freedom is two (number of levels −1 = dofj). In this experiment, the number of levels is three control factors, and the total degree of freedom is 8 (dof_T_ = *a* − 1 = m), which defines that the number of experiments using the selected orthogonal arrays should not be smaller than eight. The orthogonal array planning and the experimental data of *L*_9_(3^3^) experiment are as listed in Table 2.

**Table 2:** *L*_9_(3^3^) orthogonal arrays.

No.	Water volume (mL)	Power setting (kW)	*Pennisetum purpureum* mass(g)	Heating time (min)	*S/N* ratio (db)
1	1	1	1	27.5	−28.787
2	1	2	2	46	−33.255
3	1	3	3	55	−34.807
4	2	1	2	36	−31.126
5	2	2	3	48	−33.625
6	2	3	1	33.76	−30.568
7	3	1	3	44	−32.869
8	3	2	1	42	−32.465
9	3	3	2	38	−31.596

#### 3.1.2. Analyze Mean Value

The *S/N* ratio in the experiment can be calculated, which is then used to create a response table and plot a response diagram. The influence of the factors on the system can be calculated using the data in Table 2 to create the response table and plot the response diagram of the factors. The response value of the *S/N* ratio of each factor is calculated in Equation (2), and the calculated data are shown in Table 3.

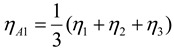
(2)
where, *η_A_*_1_ is the response value of *S/N* ratio of 1 level of factor A, *η*_1_, *η*_2_ and *η*_3_ is the response value of *S/N* ratio of No.1, No.2, No.3 respectively.

Average *S/N* for A (water volume of vessel):
average *S/N* for A1 = [(−28.787) + (−33.255) + (−34.807)]/3 = −32.28
average *S/N* for A2 = [(−31.126) + (−33.625) + (−30.568)]/3 = −31.77
average *S/N* for A3 = [(−32.869) + (−32.465) + (−31.596)]/3 = −32.31
Average *S/N* for B (heating power):
average *S/N* for B1 = [(−28.787) + (−31.126) + (−32.869)]/3 = −30.93
average *S/N* for B2 = [(−33.255)+(−33.625) + (−32.465)]/3 = −33.11
average *S/N* for B3 = [(−34.807) + (−30.568) + (−31.596)]/3 = −32.32
Average *S/N* for C (*Pennisetum purpureum*):
average *S/N* for C1 = [(−28.787) + (−30.568) + (−32.465)]/3 = −30.61
average *S/N* for C2 = [(−33.255) + (−31.126) + (−31.596)]/3 = −32.99
average *S/N* for C3 = [(−34.807) + (−33.625) + (−32.869)]/3 = −33.77

**Figure 6:**
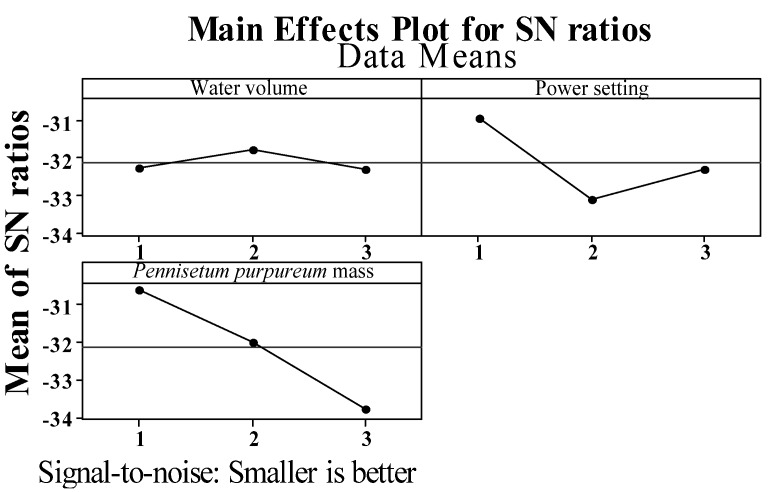
Response diagram of Taguchi experiments.

The quality characteristic is the smaller-the-better, thus, the quality characteristics are better when the experimental result of the *S/N* ratio is closer to zero. Based on the results in Table 3 and Figure 6, the optimal parameter level can be predicted as the water volume of the vessel is 150 mL, heating power is 0.5 kW, and mass of *Pennisetum purpureum* is 5 g. The optimal parameter combination of the microwave-based heating is A2 B1 C1. Based on the response in Table 3, the degree of contribution of the parameters to the system can be calculated. The calculation method is to subtract the *S/N* maximum level of each factor from the *S/N* ratio of the minimum level of each factor. The contribution degree of parameters is C > B > A.

**Table 3:** Integration results of response in Taguchi experiments.

Level	Water volume(A)	Power setting(B)	*Pennisetum purpureum* mass (C)
1	−32.28	−30.93	−30.61
2	−31.77	−33.11	−32.99
3	−32.31	−32.32	−33.77
Effect	0.54	2.18	3.16
Rank	3	2	1

#### 3.1.3. Analyze Variance

The main text paragraph Analysis of Variance (ANOVA) aims to evaluate experimental errors, and the F-ratio method is used to identify the influential degree of each factor [22]. The factor is significant when the F-ratio is greater than the smallest F-ratio in the confidence level. The factors in which the F-ratio is smaller than the minimum value are used for pooling experimental errors. The response value of the *S/N* ratio of ANOVA for each factor is calculated by the error degree of freedom (dof_e_), sum of square (SS) of variance, total SS of variance, SS of individual variance, SS of variance error, variance of variable j, error of variance, F-ratio [23]. The calculated data are shown in Table 4.

The minimum value of the F-ratio is four when the confidence level is below 80%. The values in which the F-ratio is smaller than four are pooled errors, as shown in Table 5. As can be seen, the water volume and heating power are pooled errors when the confidence level is below 80%, and the significant factor is the mass of *Pennisetum purpureum*.

**Table 4:** Analysis of Variance (ANOVA).

Source	dof	SS	V	F-ratio
Water volume	2	0.549	0.275	0.17
Heating power	2	7.362	3.681	2.25
*Pennisetum purpureum* mass	2	15.059	7.529	4.61
Error	2	3.267	1.633	–
Total	8	26.237	–	–

**Table 5:** ANOVA after pooling of errors.

Source	dof	SS	V	F-ratio
Water volume	Pooled			
Heating power	Pooled			
*Pennisetum purpureum* mass	2	15.059	7.529	4.04
Error	6	11.178	1.863	–
Total	8	26.237	–	–

#### 3.1.4. Verification Test

In the verification test, the Taguchi method applies a confidence interval to verify whether the parameter level is optimal, and determines the confidence interval of the predicted value of the optimal parameter level as well as the confidence interval of the actual value. If the two intervals overlap, the optimal parameter level proposed by the Taguchi experiment is confident.

**Figure 7:**
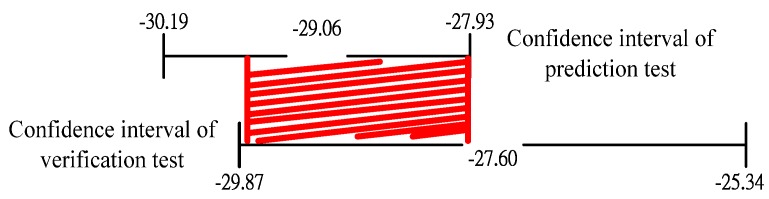
Relation between confidence intervals of predicted value and actual value.

Based on the response table and response diagram, an optimal parameter level combination can be predicted. As shown in Figure 6, the optimal parameter level combination is A2B1C1. The predicted optimal parameter level combination is tested, and the *S/N* is calculated using the results of the test. The *S/N* ratio of the optimal parameter level combination is then calculated using the equation for *S/N* the smaller-the-better. Finally, the reliability of parameter optimization is tested when the confidence level is below 80% [18,22]. An 80% confidence interval range of the experimental parameter level is presented by Equation (3); the confidence interval of the predicted value of the optimal parameter level is shown in Equation (4). The results are shown in Table 6 and Figure 7.

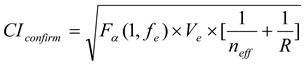
(3)

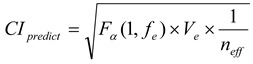
(4)
where, *α* denotes 0.2, *f_e_* denotes degree of freedom of F-ratio, *n_eff_* denotes experiment number *n*/(1 + average degree of freedom of significant factor), and *R* denotes test number, *F_α_*(1, *f_e_*) = 2.07.

**Table 6:** Comparison of predicted value and actual value of A2B1C1.

Value	*S/N* ratio	Heating time (min)
Predicted value	−29.06	27.23
Actual value	−27.60	24

Based on Table 6, the *S/N* ratio of the predicted value is −29.06, and the *S/N* ratio of the actual value is −27.60. The *S/N* of the actual value is closer to zero in each experiment, meaning that the parameter level can reduce variation. In the verification test, the confidence interval of the predicted value and the verification test overlaps, and it can be verified that A2B1C1 is the optimal parameter level combination.

### 3.2. Building Parametric Model Using System Identification

#### 3.2.1. Identification Test Design

**Figure 8:**
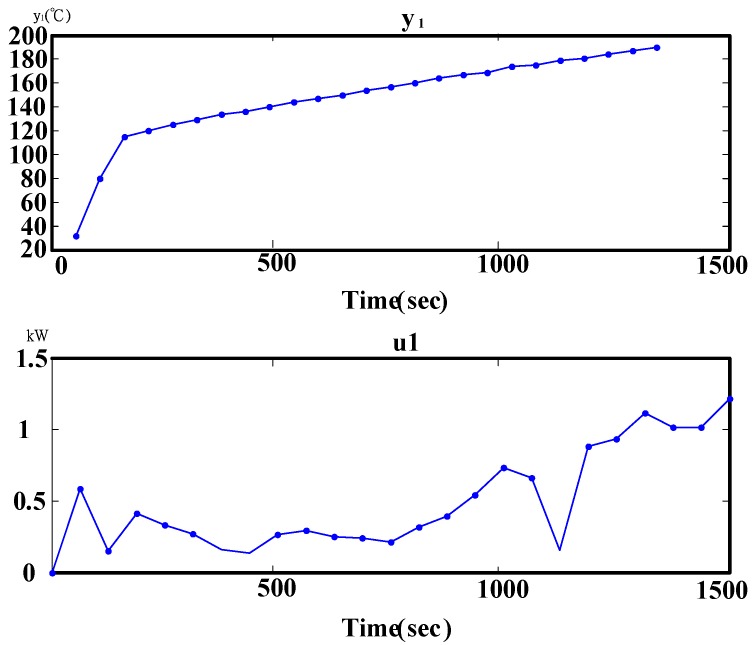
Dynamic response of actual system input and output.

The water in a closed vessel is heated in a microwave. The dynamic response of the temperature rise is directly related to the power consumption as is shown in Figure 8, thus, the consumed power and water temperature changes are used as input and output signals for the system identification. Due to the long heating time, the sampling intervals of the experiment data are one minute.

#### 3.2.2. Define System Characteristics

This paper defines the microwave-based heating system as SISO (single-input single-output), a linear time-invariant system, where the input signal and initial state of the system can determine the output signal. It is a deterministic system. The actual system is a continuous-time system; however, the samples of the system input and output signals are discrete values. For this reason, the system is defined as discrete-time system in identification. Based on the defined characteristics, a parametric model is used for system identification. The parametric model uses finite parameters to describe the dynamic characteristics of the system [20]. 

#### 3.2.3. Selection of Model Structure 

When selecting the model structure, a model approximate to the characteristics of the actual system is preferred. In addition, generated or added positions for system operations or noise measurements should be considered. If the system characteristics are not clear, several types of models can be used to identify the unknown system, and optimal identification results are used as the reference [21]. This study used ARX, OE (Output Error), BJ (Box and Jenkins), and PEM (Polynominal Error Mode) for identification.

#### 3.2.4. Selection of Model Order 

**Figure 9:**
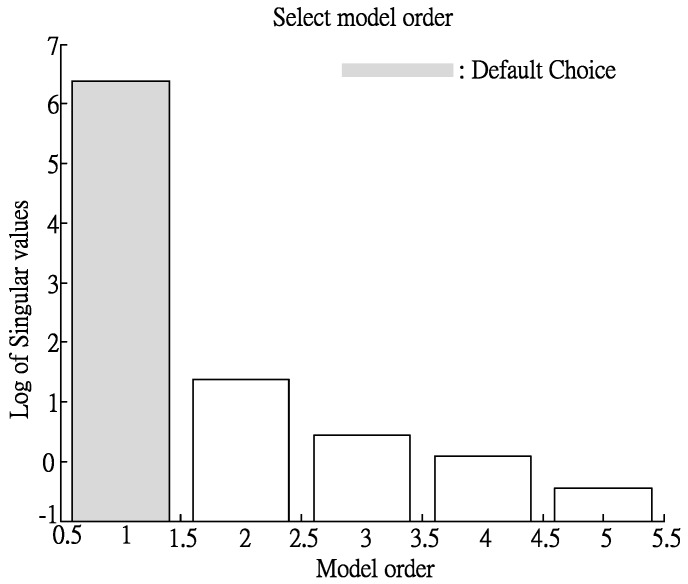
Order of optimal noise model using 4SID (SubSpace-base State Space Model Identification Method).

First, 4SID (SubSpace-base State Space Model Identification Method) is used to determine the optimal noise model order from the input and output data [20]. The 4SID is an effective system identification method used to identify a state space model using input and output data [24], where the analysis result is partial to one order, as shown in Figure 9 and the noise model order is one. Next, MATLAB is used for system identification, with the results shown in Table 7. Apart from the goodness of fit, FPE (Final Prediction Error) and Loss Function are used to identify the fitness of the model [20,21]. The smaller the values of FPE and the loss function are, the higher is the fitness. Thus, the system structure of an eight-order ARX model is selected.

**Table 7:** Identification results from the models.

Model	Order	Goodness of fit (%)	Final Prediction Error	Loss Function
ARX	7	98.84	0.059	0.211
ARX	8	99.13	0.039	0.177
OE	6	95.69	0.143	0.633
OE	7	97.31	0.042	0.335
BJ	3	98.64	0.165	0.73
PEM	1	98.2	0.875	1.39

#### 3.2.5. System Structure 

**Figure 10:**
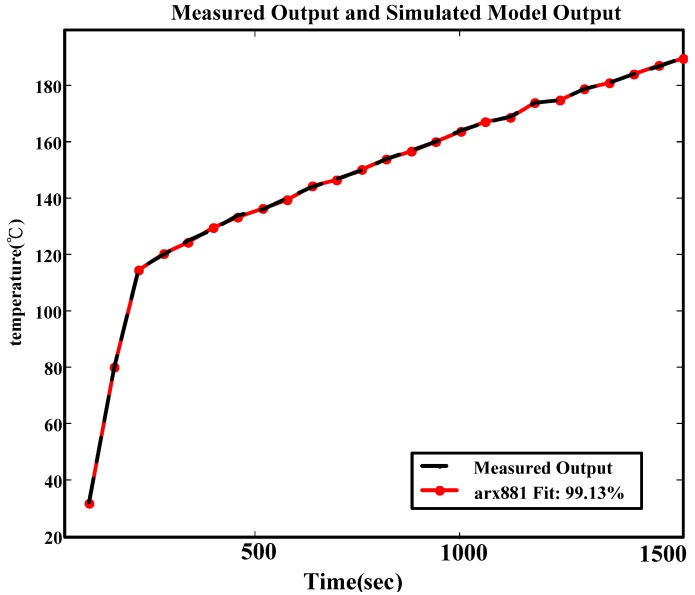
Simulated dynamic response of actual system and ARX881.

**Figure 11:**
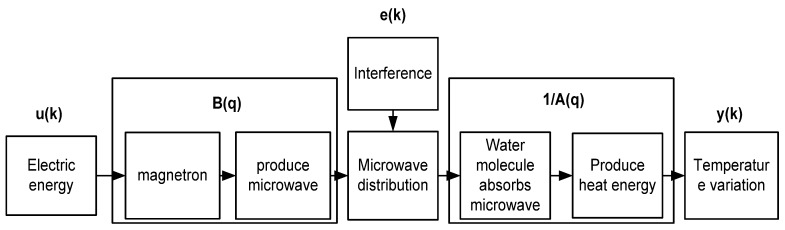
Relation between microwave energy transmission and system structure.

The energy transmission process can be used to analyze the system structure. The energy transmission of the microwave-based heating is as shown in Figure 10, where electric energy produces microwaves through a magnetron. The gain can be regarded as B(q). After microwaves are produced, their distribution in the cavity is uneven, and the heating efficiency of the microwave is affected. The factors are considered as external interference e(k), and 1/A(q) is the gain from the temperature change after the water molecules absorb microwave energy. Thus, ARX of similar structures are used for identification. The energy transmission of the microwave-based heating device and system structure are as shown in Figure 11.

#### 3.2.6. Model Construction and Verification 

**Figure 12:**
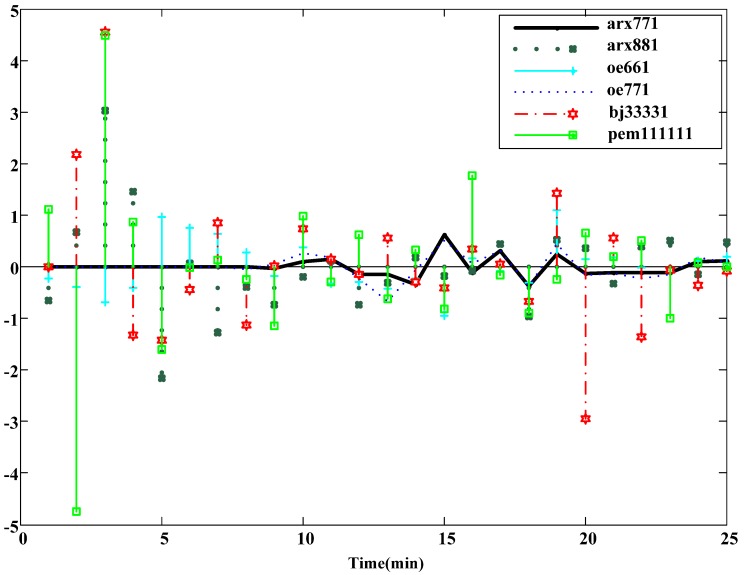
Results of residual analysis of the models.

**Figure 13:**
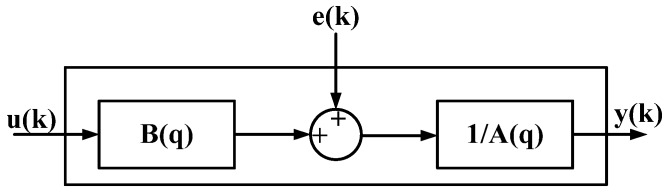
ARX block diagram.


Identification is conducted when the noise parameter of the above selected ARX in one order (ARX881), and Equation (5) can be obtained. Fit, loss function, and FPE are 99.13, 0.039, and 0.177, respectively, and the fitness of the model is very high. Next, residual analysis is conducted for ARX881 [20,21]. The result of the residual analysis for ARX881 is better that than of other models, as shown in Figure 12. The divergence is smaller, and the accuracy of ARX881 is better. In terms of fit, loss function, FPE, uncertainty, and results from the residential analysis, ARX881 is most similar to the system structure. Figure 13 shows the block diagram of ARX, and the difference equation is shown in Equation (5).

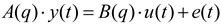
(5)
where

*A*(*q*) = 1 + 0.896*q*^−1^ − 1.116*q*^−2^ − 1.065*q*^−3^ − 0.4614*q*^−4^ + 0.3194*q*^−5^ + 0.6206*q*^−6^ − 0.3411*q*^−7^ + 1.07838*q*^−8^

*B*(*q*) = −3.162*q*^−1^ − 0.1285*q*^−2^ − 2.53*q*^−3^ − 1.255*q*^−4^ + 0.2473*q*^−5^ − 4.274*q*^−6^ + 7.583*q*^−7^ − 1.422*q*^−8^

## 4. Discussion

### 4.1. Pretreatment of Biomass Material in Steam Explosion 

(1) The data are recorded after experimentation. As shown in Figure 8, observations of the temperature rise curve can be divided into three phases, which are liquid region, liquid-gas region, and gas region. The first phase is the liquid phase, beginning from cavity heating to the cavity temperature reaching 110 °C. In this region, the water temperature is increased, but is not evaporated, and the curve presents the linear change. The experiment uses steam explosion for heating, thus, the water boiling point would increase to 100 °C. The second phase is the liquid-gas region; 10 min after the temperature reaches 110 °C. This means the critical pressure and critical temperature of the water when the water pressure and temperature are high. The third phase is the gas region, from the second phase to the target temperature of 190 °C, and the water in the cavity is gasified.

(2) As shown in Figure 5, the power curve has an irregular change when the power is not 1 kW. In future studies, the frequency conversion power will be used to adjust the input power.

### 4.2. Taguchi Experiment Planning 

(1) According to the experimental orthogonal arrays in Table 2 *L*_9_(3^3)^, the combination of different parameters has a great impact on heating time, and in the nine experiments planned by the Taguchi method, the heating time ranges from 27.5 min to 55 min, thus, the parameter optimization experiment is essential.

(2) In analysis of means, the *S/N* ratio of each experiment is calculated using a smaller-the-better equation, and Table 3—the Taguchi experiment response table and Figure 6—the Taguchi experiment response diagram, can be plotted. The analysis results are used for further analysis. It is easier to observe the effects of the control factors on the *S/N* ratio. Based on the analysis shown in Table 3 and Figure 6, the mass of *Pennisetum purpureum* has the greatest effect on the heating time, and the effect is 3.16; the water volume of the vessel has the smallest effect on the heating time, and the effect is 0.54. According to Figure 6, the combination of the predicted optimal parameters is as follows: water volume of the vessel is 150 mL (level 2); heating power is 0.5 kW (level 1); mass of *Pennisetum purpureum* is 5 g (level 1); and the heating time is reduced to 24 min.

(3) In ANOVA, the effect of each control factor on the heating time is calculated. As shown in Table 4, the control factors, the water volume of the vessel, and the heating power are insignificant factors, which are also regarded as pooled errors. Table 5 shows ANOVA after pooling of errors, and the F ratio of mass of *Pennisetum purpureum* is 4.04.

### 4.3. System Model Construction Using System Identification 

(1) Fitness of the ARX881 model is 99.13%; Loss Function and FPE are smaller than one, *i.e.*, 0.039 and 0.177, meaning that the model has a better fit.

(2) For the temperature rise curve, as shown in Figure 10, the temperature rise curve of the model and the actual system have the same characteristics. For model verification in Figure 12, the residual error of the ARX881 model is smaller than other models, and ARX is representative of the system.

## 5. Conclusions 

The Taguchi method has been used to plan the experiment design structure and to optimize the parameters. After the optimal parameter combination was determined, the system identification method was used to construct a system model. We arrived at the following conclusions:

(1) The combination of optimal parameters is as follows: water volume of the vessel is 150 mL (level 2); heating power is 0.5 kW (level 1); and the mass of *Pennisetum purpureum* is 5 g (level 1).
(2) *Pennisetum purpureum* is a key factor in pretreatment, and the F-ratio is 4.04.
(3) Black box system identification is used to construct the heating characteristic equations of the closed microwave-based heating system.
(4) The results of MATLAB indicated that an eight-order ARX881 model is representative of the system structure, and a mathematical model approximate to an actual system can be constructed.
(5) As confirmed by the results of the Taguchi method and system identification analysis, microwave-based heating can effectively increase heating efficiency and reduce pretreatment time.
